# Lectures on the Diagnosis and Treatment of Diseases of the Chest

**Published:** 1840-08-22

**Authors:** W. W. Gerhard


### MEDICAL EXAMINER.

DEVOTED TO MEDICINE, SURGERY, AND THE COLLATERAL SCIENCES.

No. 34.] PHILADELPHIA, SATURDAY, AUGUST 22, 1840. [Vol. III.

### LECTURES ON THE DIAGNOSIS AND TREATMENT OF DISEASES OF THE CHEST.

BY W. W. GERHARD, M. D.

### Lecture IX. continued.—*Bronchitis.*

The treatment is also modified by the age. In bronchitis attacking adults, bleeding from the arm is the best means of depletion,—while in children its advantages are very questionable, and it may sometimes be positively injurious. Local depletion is decidedly preferable. Children, who have passed the age of two years, may be treated by general bleeding; but, before this age, it should almost never be practised. I have very rarely found it necessary to bleed an infant suffering with disease of the chest; indeed, in these affections I only bleed as an exception. Nauseating expectorants are the remedies which have been found most generally beneficial in these affections. Among these, ipecacuanha holds the first rank: this is given in the form of a wine, or, what is still better, a syrup. When much mucus is present in the tubes, it is sometimes useful to give it in doses sufficient to produce vomiting, thus favouring the tendency which already exists: by this act the mucus is thrown off, and it is the only way in which children can rid themselves of it, as they cannot expectorate. It is generally, however, used in small doses, which act upon the skin and other secretions without producing much nausea. Antimonial wine is also used in the treatment of this affection, but I prefer the wine of ipecacuanha in the greater number of cases. The former is a more powerful remedy, and in very severe cases more reliance can be placed on it; but it is, on the other hand, more liable to induce inflammation of the mucous membrane of the stomach and intestines. Squills is also a remedy in common use in the pectoral affections of children, and is usually kept in families in the form of syrup. In cases which do not yield at once we have recourse to other remedies as adjuvants, such as mild sinapisms applied to the chest, legs, and ankles. A very good one is readily prepared by wetting a cloth with vinegar, and sprinkling it with mustard: blisters I do not consider to be so efficacious as the milder stimulants long continued. Another very important point in the management of this affection is, that the child be not suffered to remain too long on its back, as this position promotes the development of lobular pneumonia, in consequence of the mucus gravitating to the inferior portion and accumulating in the small tubes, which renders aeration imperfect, and thus favours, if it does not produce, the congestion of the lung which ends in pneumonia. The child should not be allowed to lie on its back for a longer period than two hours. This direction may appear to be trivial, but it is of much importance, for I have known death to occur from a neglect of this precaution.

As regards the quantity of blood, one or two ounces may be taken from a child under two years of age; as a general rule, an ounce for a year will answer very well. In judging of its immediate effect, you must be guided by the paleness of the patient, and not by the pulse. Sometimes a very small loss of blood produces a very decided effect upon children; therefore they should not be leeched or bled except under your immediate inspection, for cases of death, from leech-bites, have occurred among children. Cups are better than leeches, after the child has passed the age of seven years.

#### BRONCHITIS OF OLD MEN.

The bronchitis with which those advanced in age so frequently suffer, is an affection presenting much variety of form. In the first place, it varies as regards the affected portion of the bronchial tubes. It sometimes attacks the smaller tubes, and then it simulates pneumonia. Indeed, it is often called peripneumonia notha. The patient suffers excessively from dyspnœa, which is much worse when he already labours under emphysema, or any other disease of the respiratory organs, which of itself occasions a difficulty of breathing. The treatment of this affection varies according to the condition of the individual attacked by it, and the form of the disease which it assumes.

If the patient be robust, and you are called early, you will find it advantageous to resort to pretty free depletion from the arm. Great caution, however, is necessary in the use of this remedy after the disease has advanced to a certain point. When secretion has taken place, and the patient is reduced in flesh and strength, the bleeding often causes dyspnœa by preventing free expectoration. Vegetable emetics, in small doses, and expectorants, especially those of a stimulating nature, are the most valuable remedies in these cases; and in this disease you will again find that the senega is one of the best expectorants of this nature. If the patient be weak and debilitated, some carbonate of ammonia must be added to it; but the balsam of copaiba does not answer so well in this variety. If I were to select the diseases in which carbonate of ammonia is decidedly useful, I should place the bronchitis of old men and feeble subjects at the head of the list. The ammonia keeps up the strength of the patient, and promotes the natural process of cure; that is, evacuation from the bronchial mucous membrane. It therefore acts directly upon the affected part; blisters and sinapisms exercise a much more indirect influence upon it, and do good rather as revulsives in removing the inflammation, and as stimulants to the nervous system, than as direct curative agents.

In some cases of bronchitis there is a viscid secretion, with deposit of lymph, which causes great dyspnœa on account of the formation of a membrane in the tubes; sometimes this membrane has a tubular form, and these tubes have been ridiculously termed bronchial polypi. This formation I have observed more frequently in old persons than in children. It causes dyspnœa by protecting the mucous membrane of the tubes from the contact of the air, and by obstructing the passage into the air-cells. The lymph is detected by its presence in the expectoration, as well as by the orthopnœa. Emetics and expectorants, as those of a nauseant or stimulant kind, are appropriate to this disease, according as inflammation is present or not; but mercurials, which are the most efficient of all known medicines in preventing the formation of lymph, are sometimes required, if the affection be highly inflammatory.

Although acute bronchitis is in many cases an idiopathic affection, it also occurs frequently as a complication of other diseases. Almost no acute disease attended with fever is entirely exempt from it; and, as a general rule, the degree of fever is proportioned to the frequency and severity of the secondary bronchitis. It is thus an almost invariable attendant upon measles, typhoid fever, and in fact many of the exanthematous diseases. The secondary inflammation is most frequent at the same season of the year as the primary bronchitis; that is, in the early spring, and in the winter months, when febrile diseases are peculiarly liable to this complication. There are also many chronic diseases which singularly favour the development of acute bronchitis; these are diseases of the heart and of the lungs. I have already alluded to the connection of this disease with tubercles: this is the variety most difficult of recognition, but scarcely more frequent than the acute bronchitis which occurs during the course of the chronic variety, or in emphysema. In these cases the distress and difficulty of respiration are much greater than in the simple form of the disease.

The treatment of this variety of the disease is similar to that of the acute idiopathic bronchitis, and consists in the use of depletion, stimulants, expectorants, and diaphoretics. After the secretion from the mucous membrane has set in, local depletion may be used according to the necessity of the case. Cups are more beneficial than leeches, as they produce greater irritation with a smaller abstraction of blood; they are generally applied between the scapulæ. Stimulants applied externally often produce a good effect; sinapisms and other remedies of this kind are usually placed upon the anterior portion of the thorax. They act as counter-irritants.

### CHRONIC BRONCHITIS.

I have still to speak of the chronic and specific varieties of bronchitis, and shall commence with the chronic.

Chronic inflammation of the serous and mucous membranes may originate in two ways: 1st, it may be chronic from its commencement; 2dly, it may follow acute inflammation, which frequently passes into the chronic form. The latter is the more common in the case of bronchitis.

Chronic bronchitis presents several varieties: *the common chronic mucous catarrh*; *chronic catarrh, with a thin, glairy secretion*; and the *dry catarrh*, with thickening of the bronchial mucous membrane. There is another form described by some authors, viz. the *pituitary;* but this is very rarely met with so strongly characterized as to be distinguished from the second variety, or chronic catarrh with a glairy secretion, and therefore it may be considered as a mere modification of it.

The first variety, or the common mucous catarrh, is the most common. It is characterized by a secretion of white mucus, sometimes puriform, generally in irregular shreds, and but rarely moulded to the form of the tubes. It consists of mucus rendered albuminous or purulent in the progress of the inflammation. The febrile excitement in this affection is various, being sometimes very decided, but in a majority of cases comparatively mild. It is usually greater at night than during the day. The appetite, and other constitutional symptoms, vary very much.

The diagnosis is based upon the presence of certain physical and functional symptoms, and the absence of other physical signs which are found in analogous affections of the chest. The positive signs are, in the first place, the rhonchi; these are of the moist variety, and vary very much, the subcrepitant being heard at one time, and the coarse mucous rhonchus at another. The respiration is sometimes loud and rough, at others feeble; the latter state is much more common. These are the positive signs. Our diagnosis is rendered certain by the absence of signs which other diseases of the chest always present. Thus, it is distinguished from phthisis by the absence of flatness at the summit of the lungs, (which we almost always find in this affection,) and of the bronchial or cavernous respiration. Although these signs are absent in the commencement of the affection, we not unfrequently find them supervene after it has continued a certain time, as chronic bronchitis is often a precursor of phthisis. This change in the condition of the lungs is shown by constitutional as well as local signs. An increase of febrile excitement takes place, and the patient becomes more emaciated. Emaciation sometimes occurs without the supervention of phthisis, from the alimentary canal being involved, and from the febrile excitement; but this is of rare occurrence, and we scarcely ever meet in our practice with cases in which the diagnosis is rendered obscure on this account. After the tuberculous disease has taken place, it is exceedingly rare that the patient recovers. Sometimes the change that is about to take place seems to be indicated by the constitutional signs before the development of tubercles has occurred, by the febrile excitement, by the other symptoms being decidedly increased, and by a change in the complexion and countenance. This is a time when a correct diagnosis is of very great importance, as a proper plan of treatment may retard or prevent the development of a disease which is almost always fatal.

*Treatment.*—The treatment of this form of chronic bronchitis is somewhat similar to that pursued in the acute. General blood-letting is not often indicated; but the abstraction of small quantities of blood, by means of cups, often repeated, produces very good results; the cups are usually applied in the axilla, between the scapulæ and under the clavicles. If the disease at any time assumes a more acute form, general bleeding comes in very well. Leeches are sometimes used, but cups are preferable on several accounts; they produce a greater degree of irritation, without so great a loss of blood, and are cheaper and more convenient. Counter-irritants to the chest are very good adjuvants. These are numerous, and various in the degree of irritation they produce. I prefer the milder ones, such as Burgundy pitch, croton oil, &c., which being applied over a large surface produce, I think, a much better effect than blisters and tartarized antimony, which must be limited to a comparatively small portion of the chest. Liniments of a stimulating character have been much recommended; these consist of ammoniacal and terebinthinate mixtures. The noted empiric St. John Long was in the habit of treating thoracic diseases solely by applications of this character. Flannel worn next to the skin, and woolen stockings to the feet, are essential as adjuvants.

As internal remedies the stimulant expectorants should be used, except in those cases in which the disease approaches the acute form, when the antiphlogistic and sedative medicines are much more effectual. These are ipecacuanha, tartarized antimony, &c. Of these I prefer the ipecacuanha, as it is much milder in its action, and more easily borne than tartar emetic, which, after it has been employed for a few days, is apt to affect the mucous membrane of the stomach and intestines. In the more chronic cases the balsamic expectorants are employed with great advantage. Of these the balsam of copaiba is the most efficient, but it is a very disagreeable remedy, and cannot be taken by persons who are at all dyspeptic. Your success in the employment of this remedy with patients of this class depends very much upon your mode of administering it. The following formula is a very good one:

℞. Balsam Copaibæ ʒj. vel ʒij.

Tinct. Cardamom. Comp. ʒj.

Gum. Acac. q. s.

Aq. Menth. ℥vss.

M.

You should commence with half a drachm of the balsam in 24 hours, which quantity is to be gradually increased up to one or two drachms in the same period. If it produces much purging after administering it for some days, its use must be stopped, as this is an evidence that it has made an impression on the system. This remedy is only to be resorted to when others have proved ineffectual, as it is exceedingly disagreeable to the patient. There are other remedies of a milder nature, which can be taken with more facility; they are generally given in the form of syrups or lozenges. Most persons prefer the former, as they have been accustomed to the use of cough mixtures, which are generally in the form of syrup. Syrup of seneka is one of the best in the very chronic cases; syrup of ipecacuanha is also frequently used, and many prefer a combination of the two, which answers a very good purpose. I often use a combination of seneka and Prunus Virginiana, or seneka and sanguinaria, but more frequently the former. The following formula is one which I generally prescribe:

℞. Senegæ.

Prun. Virgin. } āā ℥ss.

Aq. Bullient. Oj. M.

Macera per horas xij., dein cola et adde saccharum album, q. s.

This quantity may be taken in two days, and in the management of the disease is a most effectual remedy.

Gum ammoniac is a remedy much used by some physicians, and in its action nearly resembles the balsam of copaiba. Assafœtida is also an excellent expectorant, but its taste is objectionable to many adults; it may be given in the form of lac assafœtida. For children it is peculiarly adapted.

Opium, as a remedy in bronchitis, has many advocates, and it is certainly very beneficial in some cases; but I am very cautious as regards its employment in those affections for the relief of which a secretion is necessary. I only use it as a means of procuring sleep when the cough is troublesome at night, especially when there is much irritation about the trachea and larynx.

If you prefer the form of lozenges, one of the best prescriptions will be that of the balsam of *Tolu*, which may be made into lozenges, each containing a grain of ipecacuanha, to which a small portion of morphine may be added if necessary.

The next point in the treatment is the hygienic condition under which the patient should be placed. And here the question occurs, should the patient be confined to the house or not? I would not, as a general rule, enjoin this upon him; but where there is a certain degree of acuteness in the symptoms, I think it necessary. In other cases he would lose much by keeping within doors in mild and pleasant weather, although during the cool, damp weather which is common in the spring, he should by no means expose himself. You should therefore direct your patient to take gentle exercise in the open air in good weather, unless he should find it to disagree with him.

A sea voyage to a warmer climate will often remove a bronchitis of long-standing; but it is often very inconvenient for the patient, and in many cases it is not in his power to try it. In proportion as the disease becomes more and more chronic, the patient may increase the amount of exercise, and endeavour to stimulate the muscles and exterior, and thus produce a general but mild revulsion from the interior organs. This treatment is not only of great service in removing the bronchitis, but it is the best means of obviating the danger of pulmonary phthisis. The rules as to clothing and warmth are obvious enough: the great secret of the treatment consists in diffusing the action and nutrition throughout the muscular and tegumentary tissues, and thus giving to the bronchial mucous membrane an opportunity of regaining its normal condition. The medicinal treatment is more complex; but if you separate it from the hygienic management, you will find it less efficacious than the latter.

The second variety of chronic bronchitis resembles the *pituitary* catarrh of Laennec. It is distinguished from the preceding by several peculiarities. It does not usually follow the acute affection, but commences with its peculiar characteristics. It generally occurs at irregular periods; but in many individuals it takes place at regular seasons; in this climate usually at the close of the summer, about the month of August. It is quite frequent too in Great Britain.

The local signs of this affection consist of the various rhonchi, both dry and moist, the latter being found usually at the lower part of the chest, the former in the upper portion; there is, however, a predominance of the moist rhonchi over the dry, and of the sibilant and subcrepitant over the coarser varieties, as the smaller tubes are more affected than the larger. Sometimes all the rhonchi are heard at once, and produce a singular confusion of sounds, to which Laennec has applied the term *cantus omnium avium*. In some cases the air-cells are dilated, which renders the respiration feeble, and gives rise to much dyspnœa, resembling asthma, and indeed it may be set down as one of the varieties of this disease. The dyspnœa complicating the affection, however, more frequently arises from thickening of the tubes preventing the passage of the air into the vesicles. These attacks of dyspnœa are sometimes permanent, sometimes transitory. The fever attending this variety of bronchitis is very slight, and there is very little emaciation.

When this disorder assumes a periodical character, and occurs at a particular period, it lasts several weeks, and in general cannot be cut short by treatment. The duration of this variety of the disease is less than that which occurs at irregular intervals, and it resembles in many respects the more ordinary forms of acute catarrh, but is much more intractable.

*Treatment.*—Bleeding in a majority of cases is not well borne; but when the symptoms are acute, it may be prescribed with advantage. The remedies to be used are those which are calculated to relieve the dyspnœa. These are principally the nauseating expectorants, of which I think lobelia to be decidedly the best, so given as to produce slight nausea; it thus favours secretion and expectoration. Balsam of copaiba is also a very good remedy; but the same objections apply here as in the other forms of bronchitis. Venetian turpentine has been very much used, and is an excellent remedy.

In the periodical form of the affection, after the paroxysm has commenced, no treatment has yet succeeded in cutting it short. There is, however, one point which demands our attention, viz. the prevention of the occurrence of the paroxysm. In one case for which I prescribed cold affusions and the exhibition of quinine, previously to the attack, the disease appeared much later than usual, was milder in its character, and its duration was much less.

*Dry Catarrh.*—The third variety is perhaps as frequent as either of the others, and is by a strange contradiction in terms called *dry catarrh*, because there is little or no expectoration, differing in this respect from the other varieties. The prominent lesion in this form of bronchitis is a thickening of the mucous membrane. This, though rendered evident by the local signs, is not always found after death: in this respect it is analogous to other congestions of membranes. It is attended with very little febrile excitement; and the functions of the alimentary canal are but slightly, if at all impaired. The cough is short and dry, thus differing from the cough which attends the other varieties, the latter being loose. The chest is sonorous throughout, and in some cases preternaturally so, on account of the emphysema, which is a frequent attendant. The respiration is generally feeble, and sometimes a rough rustling sound is heard, arising from the friction of the air-cells against the pleura. The dry rhonchi are usually heard, though not in all cases, as the thickening must proceed to a certain point in order to produce them; they, of course, vary according to the particular part of the bronchial tube which is affected. But, it generally occurs that the sibilant rhonchus is chiefly confined to the anterior part of the chest, and the sonorous rhonchus to the neighbourhood of the larger tubes. Besides emphysema, there is another complication which is frequently met with, and which, like it, is produced by the violent efforts made in coughing,—I allude to hypertrophy and dilatation of the heart. These three affections frequently coincide; and the heart disease, the dry catarrh, and emphysema, form a triple lesion. The duration of this variety of chronic bronchitis is greater than that of the other two. It continues to an indefinite period,—the patient often labouring under it for several years, unless some acute affection of the lungs should supervene, which is then rendered more grave by the previous existence of the dry catarrh. When, for instance, pneumonia attacks a person who is affected with dry catarrh, the dyspnœa which, under ordinary circumstances, attends the acute affection, is rendered more severe by the existence of the chronic: this, of course, renders our prognosis much more unfavourable than it is when the disease is not complicated with an acute inflammation, or when the dyspnœa is not severe.

*Treatment.*—Very little advantage results, I think, from the employment of medicines in this variety of chronic bronchitis. It is, however, of importance to attend to the hygienic condition of the patient. His clothing should be warm, and his chest and extremities protected by flannel; and he should not expose himself in damp and inclement weather, while he should take exercise when the weather is dry and pleasant. The patient, however, sometimes insists upon having medicine, and it is as well to gratify him in this respect. The balsams and turpentines have been much used; also alkalies, which are highly recommended by Laennec.

I have not spoken of the use of mercurials in the treatment of chronic bronchitis. They have been used from time to time; but the results have not been such as, in my mind, to warrant their employment.

There is an affection which resembles very much the dry catarrh, that is, the cough which occurs in some cases of dyspepsia; it is usually dry, and sometimes attended with rhonchi, although in general they are not heard. The diagnosis here depends upon our knowledge of the previous affection of the stomach. In other cases, however, a bronchitis, previously existing, is aggravated by the occurrence of an affection of the stomach: here the priority of symptoms must be your guide. We can generally succeed in arresting this cough by the use of tonics, alkalies, and other remedies adapted to the state of the stomach.

Chronic bronchitis may arise from a variety of causes, as a fever, an acute attack of disease of the lungs, &c. It is frequently found co-existing with tuberculous phthisis, which may either have preceded or followed it; and it may follow any other disease of the lungs, or it may be the cause of such affection. Indeed, we seldom meet with a disease of the parenchyma of the lungs unaccompanied by bronchitis, which we might naturally suppose would be the case, since the bronchial tubes constitute so large a portion of the respiratory organs. The disease receives the name of *bronchitis* when the affection is confined to the tubes; when the parenchyma is attacked, the bronchitis is looked upon as a mere complication of the more serious affections, and the designation of the disease accords with the principal lesion. This rule should be adhered to, otherwise you will confound together many different affections, and may include phthisis, laryngitis, and pneumonia, under the common designation of bronchitis.

### PECULIAR VARIETIES.

Besides the modifications of bronchitis which depend upon the duration of the disease, and the age or other peculiarities of the individual, there are other varieties which are specific in their character, and depend upon a peculiar condition of the system, produced by a constitutional disorder. Of these varieties one of the most frequent is *pertussis*, or *whooping cough.* This is an affection of the nervous system accompanied by bronchitis, in which sometimes the one, sometimes the other predominates; the affection of the nervous system being in some cases very severe, with but little cough, whereas the cough is frequently very bad, with comparatively slight nervous symptoms. We almost always meet with this disease in children, though adults are occasionally attacked by it. It is a self-limited disease, and therefore cannot be cut short by treatment, although its complications may be removed or palliated. Though the inflammation of the bronchial tubes is merely the local part of the disease, yet it is in one sense the most important, for patients generally die of the bronchitis and its immediate effects. The secretion from the mucous membrane is much greater than in ordinary varieties of bronchitis; and in children it tends constantly to accumulate in the inferior parts of the tubes: they are in this way gradually enlarged until permanent dilatation results. The thickening and congestion of the mucous membrane do not differ from the same alterations in ordinary bronchitis. When a fatal termination occurs, it generally arises from the feebleness of the patient, and a consequent inability to expectorate, or as is the case with children, to discharge the secretions by vomiting.

The parenchyma of the lungs may become congested and inflamed, producing a pneumonia which may prove fatal.

The principal sign of this disease is the peculiar whooping character of the inspiration: this is caused by the forcible expulsion of air from the chest, in fits of coughing, and sometimes occurs in other forms of bronchitis, which, however, do not often possess the paroxysmal character of pertussis. In addition to the cough we meet with the rhonchi, both dry and moist, and very often with a gurgling caused by the collection of fluid in the dilated bronchi. The cough usually lasts for several weeks; it then declines gradually, and the rhonchi disappear. It is gradual in its attack, being at first slight, and then becoming violent. It comes on in paroxysms, of which, in mild cases, there are usually five or six during the day, the patient being free from cough in the interval. In severe cases the number of paroxysms is much greater. They sometimes occur as often as once an hour, and occasionally there is only an interval of a few minutes. In such cases the patient generally dies of exhaustion. The secretion in the bronchial tubes consists of thick, glairy mucus; when it has continued for a long time, it sometimes contains a small portion of pus, intermixed with blood. Sometimes blood is effused, and a partial hæmoptysis occurs. The secretion is usually thrown off by vomiting, especially in young children, who cannot expectotorate. The appearance of the face in this disease is peculiar, being of a bluish colour, accompanied by puffing of the eyelids. This is the effect of the violent efforts made in coughing, and the congestion consequent upon them. It is in some degree a measure of the severity of the affection.

When fever occurs it indicates the existence of inflammation of the lungs, and when high, is a symptom of much gravity. When the development of tubercles takes place towards the close of the disease, the fever continues with a quick, irritable pulse. It is usually the miliary form of tubercles which occurs under these circumstances, and is almost always fatal.

The *diagnosis* is pretty clear after the second week: the paroxysmal character of the cough, with its whooping inspiration, its complete intermission, and the recurrence of the paroxysm during any disturbance of the mind, are sufficient to characterize it.

The *prognosis* is generally favourable in the simple forms of the disease, but becomes less so in proportion to the severity of the complications.

*Treatment.*—As the disease cannot, as a general rule, be arrested, we should palliate its symptoms, and assist nature in the means which she has pointed out for its relief, we should therefore promote the secretion in the tubes, and favour its removal. Therefore we should employ mild emetics, which tend to bring about both these ends. They should be given once or twice a day for a week or two, In this affection there is always a disposition to vomit; and as this action, brought on by artificial means, is milder than when it occurs spontaneously, emetics afford very great relief. After this treatment has been continued for the time above specified, we should make use of remedies whose action is slower but analogous to that of emetics, for this is the means pointed out by nature for the cure of the disease; and it is a maxim in therapeutics, that when a secretion is intended by nature to remove any diseased state of the economy, we should favour or moderate it, and not arrest it. Ipecacuanha, in the usual expectorant doses, may be used for this purpose, and answers very well,—but one of the best remedies in this affection is assafœtida, as it favours expectoration, and also controls the disorder of the nervous system, which constitutes so large a part of the disease. It may be given to children of eight or ten years, in doses of two or three grains, several times daily. However, it cannot always be given internally, as it is so repulsive to the senses; applied externally, in the form of a plaster, it acts very well, producing an impression on the nervous system, and moderating the paroxysms. Ammoniac, galbanum, &c., are used in the same manner. Revulsives to the chest are useful, but not always necessary; when required, I prefer sinapisms to blisters or moxas. There is another remedy which is much more powerful than these,—that is, the extract of belladonna; I know of no practitioner who uses it more boldly, or with better effect, than Dr. H. Corson, who resides not far from this city. I regret that I have not his formula at present. Still, you cannot be too cautious in the administration of this medicine, which is certainly always attended with some risk. The success which attends its administration in whooping cough, is stated to be greater than that of any other remedy.

The clothing should be warm, flannel to the chest, &c.

The complications are various affections of the lungs, which are, when very acute, to be treated by general and local blood-letting, and other remedies required in the affections occurring idiopathically. Phthisis occurs as a sequela of this disease, and does not require medication; it is best treated by a change of air, which is advantageous in the declining stages of all severe cases of pertussis.

As pertussis rarely occurs with adults, we are apt to make an incorrect diagnosis when it does thus occur: this should be borne in mind, as we might confound it with a variety of bronchitis resembling pertussis, which is exceedingly difficult to get rid of. Ordinary bronchitis may be complicated with the nervous spasm; but the disease should not be confounded with pertussis, unless the spasms are disproportioned to the bronchial affection. This constitutes the peculiarity of the disease, and gives to it that mysterious difference between it and other varieties of bronchial inflammation.

BRONCHITIS DEPENDENT UPON A CONSTITUTIONAL TAINT.

There are certain cases of bronchitis which depend on a particular diathesis, or a peculiar condition of the system induced by a specific affection: to this class belong the syphilitic and scrofulous bronchitis. But you will sometimes find that the syphilitic variety is singularly similar to phthisis in the emaciation and other constitutional symptoms; so much so that the deterioration of the health is such as to end in phthisis. The scrofulous bronchitis is attended with a very abundant secretion of a thick, glairy mucus, and is in most cases complicated with an inflammation of the upper portion of the respiratory tubes, so that the nasal cavities are sometimes more affected than the bronchi; it must be treated with remedies calculated to correct the morbid state of the system, such as mercury, iodine, sarsaparilla, for the syphilitic variety; iodine, iodide of iron, and other chalybeates, may be used in scrofulous disease, besides resorting to local remedies.

*General Remarks.*—Although bronchitis, as a disease, presents many varied characters, yet there are certain features which are common to every form of it. In all, the turgescence of the bronchial mucous membrane with blood gives rise to the chief difficulties in the respiration, and, when this congestion extends to the smaller tubes, the dyspnœa becomes excessive, and may be a source of immediate danger. This simple congestion of the membrane occurs in the early period of acute cases, and in the dry catarrh it becomes a chronic condition, and lasts for an indefinite period. The most easy and frequent termination of the congestion is by direct secretion from the bronchial tubes; that is, by the formation of a mucous and muco-purulent discharge; but in many cases the general circulation may be restored, and the congestion removed, by the free discharge from the capillaries of the skin, or some other tissue. If this relief does not follow, the tendency of all cases of dry bronchitis is to congest the heart, and to distend the vesicles of the lung; hence emphysema of the lungs, and dilatation the heart, frequently depends upon this cause.

The other varieties of bronchitis, whether acute or chronic, are those in which secretion takes place; if this secretion be of a natural, healthy kind, the inflammation ceases; thus the thin albuminous secretions are replaced by a more consistent mucous or muco-purulent expectoration, which again gradually passes into a more transparent mucous discharge, which gradually ceases. But, although this is the course of the disease when it terminates favourably, in many cases the secretion of mucous and muco-purulent matter will continue, while the inflammation does not abate. These are the chronic cases of mucous catarrh. In this variety the discharge is analogous to what takes place in chronic dysentery, when the inflammation is not relieved by the secretion. The difference appears to arise from a modification in the mucous tissue, by which the vessels remain permanently enlarged, and recover with difficulty, unless a stimulant is administered which should excite this new action. Hence we use what are called the stimulating expectorants so largely in these forms of bronchitis; these remedies supply the excitement necessary to the relief of the disease by a new and more healthful secretion. The inhalation of the vapour of water, of tar, ether, &c., act much in the same way, but are more direct stimulants of the membrane. The depleting remedies, which are often necessary in severe bronchitis, act, of course, very differently from the stimulating expectorants; they merely equalize the circulation of the bronchial vessels, and thus lead towards health by removing the vascular excitement which keeps up the disease. The results of this mode of treatment will, of course, be essentially the same with those derived from the stimulating expectorants, but the *modus operandi* is totally different. The revulsive means are more analogous to the direct antiphlogistic remedies, and produce very nearly the same effects.

Bronchitis is therefore a multiform disease and varies both in symptoms and treatment with almost every modification of the body; it may be highly inflammatory, and require the most vigorous depletory means, or it may degenerate into a mere chronic oozing of mucus from the vessels. The object of the physician is to vary his treatment according to these different conditions, and, at one time, to resort to vigorous antiphlogistic measures, and, at another, to a course of treatment which is totally different. I have laid much stress upon the latter practice, because it is suited to a greater number of cases, but I am not the less convinced that in the cases which are decidedly inflammatory, the most effectual relief is produced by the antiphlogistic practice; it may afterwards be followed by any other remedies that the case may seem to require.

